# Expression and Role of Biosynthetic, Transporter, Receptor, and Responsive Genes for Auxin Signaling during Clubroot Disease Development

**DOI:** 10.3390/ijms21155554

**Published:** 2020-08-03

**Authors:** Arif Hasan Khan Robin, Gopal Saha, Rawnak Laila, Jong-In Park, Hoy-Taek Kim, Ill-Sup Nou

**Affiliations:** 1 Department of Horticulture, Sunchon National University, Suncheon 57922, Korea; gpb21bau@bau.edu.bd (A.H.K.R.); gopalagr@pstu.ac.bd (G.S.); rawnak@bau.edu.bd (R.L.); jipark@sunchon.ac.kr (J.-I.P.); htkim@sunchon.ac.kr (H.-T.K.); 2 Department of Genetics and Plant Breeding, Bangladesh Agricultural University, Mymensingh 02202, Bangladesh; 3 Department of Agronomy, Patuakhali Science and Technology University, Patuakhali 8602, Bangladesh

**Keywords:** Chinese cabbage, auxin, clubroot, *Plasmodiophora brassicae*, expression analysis

## Abstract

Auxins play a pivotal role in clubroot development caused by the obligate biotroph *Plasmodiophora brassicae.* In this study, we investigated the pattern of expression of 23 genes related to auxin biosynthesis, reception, and transport in Chinese cabbage (*Brassica rapa*) after inoculation with *P. brassicae*. The predicted proteins identified, based on the 23 selected auxin-related genes, were from protein kinase, receptor kinase, auxin responsive, auxin efflux carrier, transcriptional regulator, and the auxin-repressed protein family. These proteins differed in amino acids residue, molecular weights, isoelectric points, chromosomal location, and subcellular localization. Leaf and root tissues showed dynamic and organ-specific variation in expression of auxin-related genes. The *BrGH3.3* gene, involved in auxin signaling, exhibited 84.4-fold increase in expression in root tissues compared to leaf tissues as an average of all samples. This gene accounted for 4.8-, 2.6-, and 5.1-fold higher expression at 3, 14, and 28 days post inoculation (dpi) in the inoculated root tissues compared to mock-treated roots. *BrNIT1,* an auxin signaling gene, and *BrPIN1,* an auxin transporter, were remarkably induced during both cortex infection at 14 dpi and gall formation at 28 dpi. *BrDCK1,* an auxin receptor, was upregulated during cortex infection at 14 dpi. The *BrLAX1* gene, associated with root hair development, was induced at 1 dpi in infected roots, indicating its importance in primary infection. More interestingly, a significantly higher expression of *BrARP1*, an auxin-repressed gene, at both the primary and secondary phases of infection indicated a dynamic response of the host plant towards its resistance against *P. brassicae*. The results of this study improve our current understanding of the role of auxin-related genes in clubroot disease development.

## 1. Introduction

**Figure 1:**
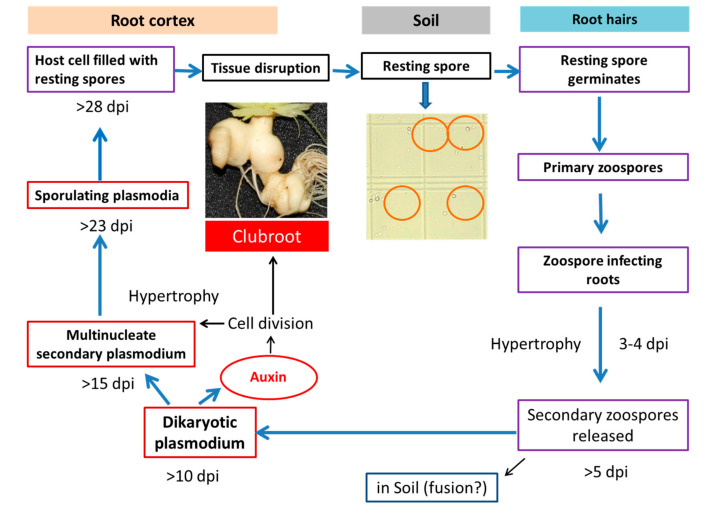
Involvement of auxin in clubroot formation during the life cycle of *Plasmodiophora brassicae* (Robin et al. [9] after Dekhuijzen [10]; Müller and Hilgenberg [11]).

Clubroot, a serious disease of Brassicaceae family members throughout the world, is caused by an obligate biotroph *Plasmodiophora brassicae* Woronin. The “clubroot” is symptomatically known by enlarged roots, which eventually results in wilted plants in their upper parts due to nutrient and water losses at the later stages of the disease [1]. *P. brassicae* completes its life cycle in two phases: a primary phase which is mainly confined to root hairs of the host plants and a secondary phase that takes place in the cortex and the stele of the hypocotyl and roots of the infected plants, leading to cell hypertrophy (cell division) and hyperplasia (cell elongation) and resulting in “gall” or “club” formation (Figure 1) [2,3,4]. Cell division was reported to start from 4 days after inoculation (dai), which continues throughout the secondary cycle of infection, resulting in hypertrophied cells large in size that harbor secondary plasmodia and resting spores [5]. These resting spores are highly persistent, and they may remain infectious up to 15 years in soil [6]. After completion of the secondary phase, the galls in the infected plants can be ten times larger than the noninfected cells [7]. The infection process alters the metabolism and changes the hormone pools of the host [8]. In the progression of a clubroot disease, pathogens can efficiently alter the auxin responses [8].

Auxins are well-known phytohormones for their pivotal role in plant development and defense responses [1,12]. They influence cell division, enlargement, differentiation, and polarity. At the whole plant level, they are involved in regulating tropisms, apical dominance, and root initiation. Intriguingly, auxins have also been reported to regulate various processes related to growth of pathogens and symbionts [13]. Auxin signaling is an important aspect that plays a central role in many plant–pathogen interactions [14]. There are so many cross-talks on auxin signaling and conjugating auxin pathways in the development of clubroot disease [5,7,15,16,17,18]. After the infection of *P. brassicae*, the cell division process concomitantly involves activities of auxins, cytokinins, and brassinosteroids [19,20,21]. Cell-to-cell auxin transport is very crucial for early clubroot symptom development [3]. Cell division, cell elongation, and eventually the production of hypertrophied cells during clubroot development appears to be exclusively related to higher auxin availability and biosynthesis [3,5,21,22,23,24]. For example, in Chinese cabbage (*Brassica rapa*), an upregulation of the auxin inducible genes viz. *nitrilases* and *myrosinases* via the indole-3-acetonitrile (IAN) pathway indicated their vital role in maintaining higher auxin content in cells during clubroot formation and expansion between 28 and 42 days after inoculation (dai) [25]. The mutants of auxin biosynthesis gene *“nitrilase”* in *Arabidopsis thaliana* not only delayed gall formation but also reduced the gall size after inoculation of *P. brassicae*, indicating the pivotal role of auxin in clubroot formation [17,23]. In addition, morphological changes in the roots including cell elongation after the infection with *P. brassicae* at 6 dai coincided with the elevated amount of indole-3-acetic acid (IAA) and stimulation of auxin-inducible genes in roots [3].

In spite of the well-established significance of auxins in clubroot disease development, the fluctuation of contents of IAA, INA, and other auxins did not match the infection level in *B. rapa* roots [26]. Auxin biosynthesis-related genes exhibited a wide range of variability in transcript shifts between clubroot-infected and noninfected root tissues in *A. thaliana* in the literature. Microarray data on early phase of clubroot infection at 4, 7, and 10 dai demonstrated significant induction or suppression of relatively lower number of genes in *A. thaliana* [27]. Microarray analysis using Laser Microdissection and Pressure Catapulting (LMPC) of *P. brassicae* infected and noninfected roots of *A. thaliana* demonstrated differential regulation of 66 genes associated with auxin metabolism and signaling [21]. More recently, RNAseq data of two contrasting Chinese cabbage lines at 30 days post inoculation with *P. brassicae* revealed differentially expressed 188 and 138 genes, respectively related to plant–pathogen interaction and hormone signal transduction [28]. By contrast, transcriptome analysis during infection at the root cortex and the clubroot-infected and noninfected roots showed differential expression for more than 1000 genes at both 10 and 23 days after inoculation [20].

Several reports show that unbalanced auxin is conducive for the pathogens in favor of symptom development. To remain healthy and noninfected, a tight control over the auxin concentration by the plants is important, and it can be achieved by biosynthesis, degradation, transport, or reversible inactivation of auxin [13]. However, to date, there is no comprehensive study on the expression of genes related to auxin biosynthesis, transport, and receptor in *B. rapa* at both the primary and secondary phases of clubroot infection. In a recent study, Laila et al. [29] showed an association between expression of cytokinin-related genes and cytokinin contents from the published data at both the primary and secondary phases of infection in *A. thaliana* and *Brassica spp*. In this study, we have characterized 46 auxin signaling, transporter, receptor, and responsive genes in *B. rapa* through a rigorous in silico analysis. Subsequently, we validated 23 *B. rapa* auxin-related genes during primary infection, gall initiation, and gall expansion through an extensive expression study in Chinese cabbage, an important vegetable crop in southeast Asia, after inoculating a highly virulent Korean *P. brassicae* isolate. Finally, we correlated increased expression of auxin biosynthesis-related genes and simultaneous increase/decrease of auxin contents in the published data at the adjacent time-points in the clubroot-infected root tissues to denote the role of auxin-related genes. We believe that our transcript dataset on auxin-related genes helps to understand the pattern of auxin metabolism, transport, and signaling in roots and shoots of Chinese cabbage upon clubroot infection.

## 2. Results

### 2.1. Properties of Auxin-Related Proteins

In silico analysis showed that the 23 selected auxin-related genes were from different protein families like protein kinase, receptor kinase, the auxin responsive family, auxin efflux carrier, transcriptional regulator, auxin repressed protein, etc. (Table S1). The isoelectric point of an auxin-responsive GH3 family protein, except BrGH3.2, nitrilase, and aluminum-induced proteins, (BrARG10-like) are < 7.0. Small auxin-up RNA (SAUR)-like auxin-responsive protein family member BrSAUR4 has the highest isoelectric point, 10.96, followed by auxin repressed protein, BrARP1 (Table 1). SAUR-like auxin-responsive protein family members have lower molecular weights (12.03–19.75 kDa), whereas auxin-responsive GH3 family proteins have higher molecular weights (66.03–69.39 kDa). The auxin efflux carrier family protein BrPIN1 is the longest protein with 618 amino acids, whereas the SAUR-like auxin-responsive protein family member, BrSAUR1.1, is the shortest protein with only 104 amino acids (Table 1). Auxin-responsive GH3 family proteins have 587–612 amino acids (Table 1). Auxin-biosynthesis related proteins were predicted to localize in diverse cellular components including nucleus, mitochondrion, cytoplasm, cell membrane, etc. (Table 1).

### 2.2. Variation in Expression between Leaves and Roots

**Figure 2:**
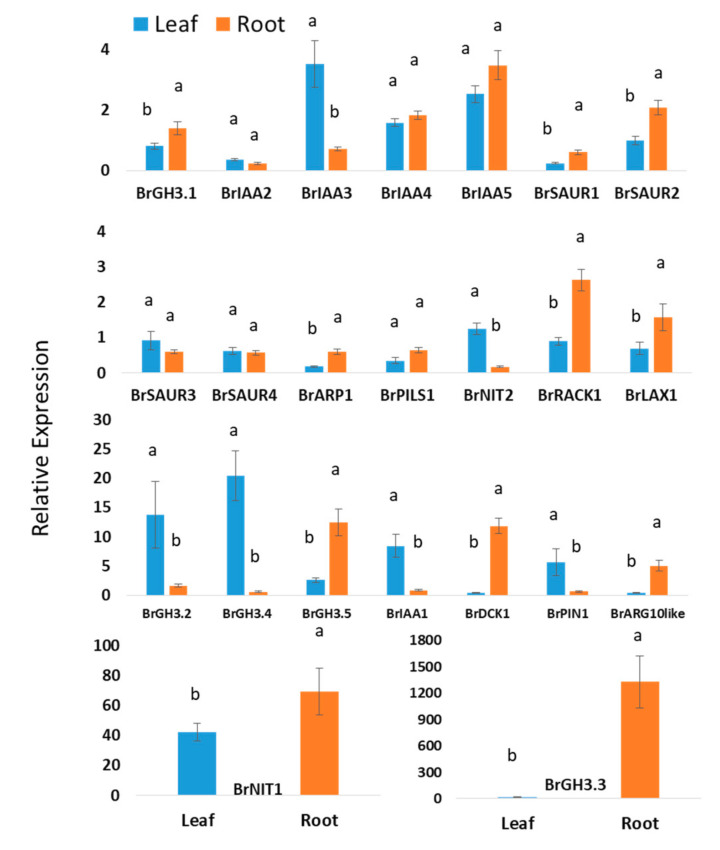
Relative expression level differences in auxin-biosynthesis related genes between leaf and root samples: Each data point represents an average value of Seosan-inoculated plus mock-treated samples of all five time-points, i.e., an average of 30 samples. The letters “a” and “b” denote statistically significant differences between leaves and roots at the 5% level of significance. Vertical bars represent standard errors. Expression of genes in leaf tissues of control (nontreated) plants on day 0 were set to 1.

Experimental data of this study revealed that nineteen auxin biosynthesis-related genes showed statistically significant difference in expression levels in between leaf and root tissues when means values of leaf and roots of all samples from two treatments and five time-points were compared (Figure 2). Five auxin signaling-related genes—*BrIAA4*, *BrIAA5, BrSAUR3*, *BrSAUR4*, and *BrPILS1*—showed nonsignificant variations between leaf and root tissues (Figure 2). *BrGH3.4*, *BrGH3.2, BrIAA1, BrPIN1*, and *BrNIT2* genes exhibited 33-, 8.4-, 9.7-, 8.3-, and 7.2-folds higher relative expression in leaf samples compared to root samples, respectively (Figure 2). *BrIAA2* and *BrIAA3* also showed significantly higher relative expression in leaf tissues compared to root tissues (Figure 2). *BrGH3.3* showed a strikingly 1328-fold higher relative expression in root tissues compared to nontreated samples (control) at day 0 (Figure 2). Relative expression of this gene in root samples is 84.4-fold higher compared to leaf samples. *BrDCK1* and *BrARG10like* genes exhibited 28.2- and 10.9-fold higher expression in root tissues compared to leaf tissues, respectively (Figure 2). Nine genes—namely, *BrGH3.1*, *BrSAUR1*, *BrSAUR2*, *BrARP1*, *BrPILS1*, *BrRACK1*, *BrLAX1*, *BrGH3.5*, and *BrNIT1*—also showed significantly higher expression levels in root samples compared to leaf samples (Figure 2).

### 2.3. Expression Profiles of Auxin-Responsive GH3 Family and BrDCK1 Genes

**Figure 3:**
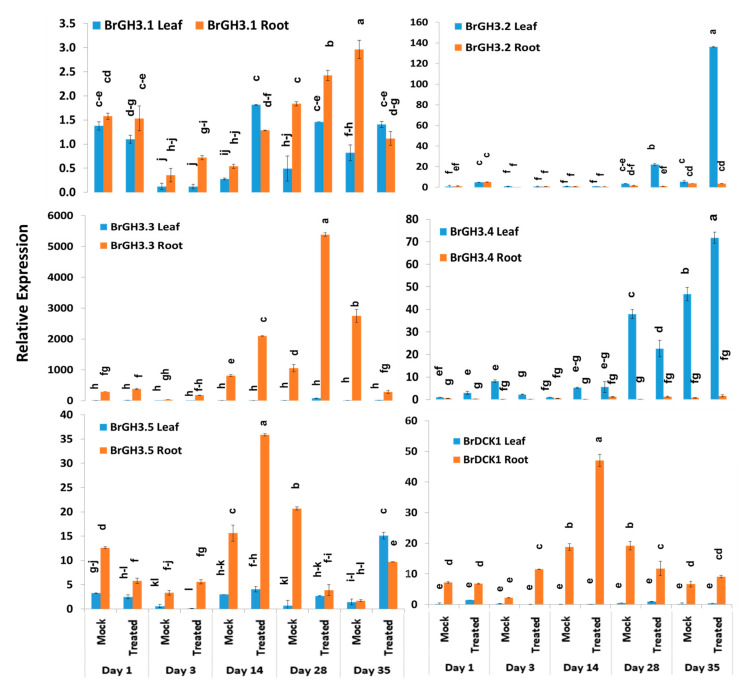
Variation in relative expression levels of *BrGH3* and *BrDCK1* auxin-related genes in leaf and root samples under different treatment × time-point combinations in mock-treated and Seosan-inoculated plants: Data represent relative expression levels of three different treatment types—sample type (leaf vs. root), treatment (Seosan-inoculated vs. mock-treated), and time-points. Vertical bars represent standard deviation. Each data point estimates the average of three independent biological replicates. Data bars separated by different letters indicate 5% statistical significance level according to Tukey’s pairwise comparisons. Expression of genes in leaf tissues of control (nontreated) plants on day 0 were set to 1.

**Figure 4:**
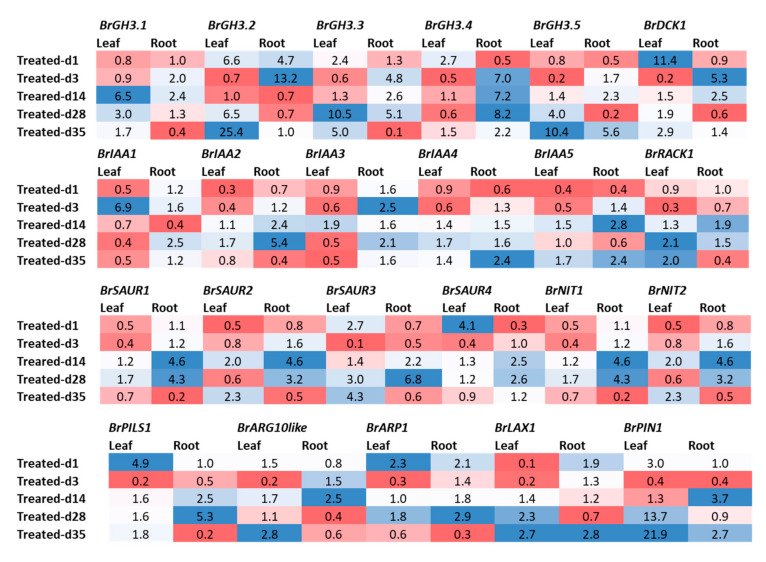
Fold changes in relative expression levels of auxin-related genes in Seosan-inoculated plants compared to mock-treated leaf and root samples at the same time point: Blue and red colors represent upregulation and downregulation of genes, respectively.

Relative expression analysis of genes showed that five auxin-responsive *GH3* family genes accounted for significant variations in time-points, treatment within time-points, and sample types (leaf vs. root) within treatment and time-points (Table 2). The *BrGH3.1* gene exhibited the highest expression level in mock-treated roots at day 3 followed by Seosan-inoculated roots at day 28 (Figure 3). Leaf and root samples of both mock and Seosan-inoculated plants showed comparatively lower expression of *BrGH3.1* gene at day 1 (Figure 3). The *BrGH3.2* gene was induced 25.4- and 6.5-fold in leaf samples of Seosan-inoculated plants at day 35 and day 28, respectively, compared to mock-treated plants (Figure 3 and Figure 4). This gene showed 13.2-fold increase in expression in root tissues of Seosan-inoculated plants compared to mock-treated plants at day 3 (Figure 4). Relative expression levels of *BrGH3.3* gene in root tissues of Seosan-inoculated plants at day 14 and day 28 were remarkable (Figure 3). The *BrGH3.3* gene exhibited high expression in root tissues. This gene showed 2.6-fold and 5.1-fold increases in expression in root tissues of Seosan-inoculated plants at day 14 and day 28, respectively, compared to mock-treated plants (Figure 3 and Figure 4). By contrast, the *BrGH3.4* gene showed remarkably higher expression in leaf tissues (Figure 3). However, this gene showed >7.0-fold increase in expression in root tissues of Seosan-inoculated plants at day 3, day 14 and day 28 compared to mock-treated plants (Figure 4). The *BrGH3.5* gene exhibited the highest expression level in root tissues of Seosan-inoculated plants at day 14 and this expression was 2.3-fold higher than mock-treated root samples (Figure 3 and Figure 4). Similar to the *BrGH3.3* gene, the *BrDCK1* gene was highly expressed in root tissues at all time-points compared to leaf tissues (Figure 3). The *BrDCK1* gene exhibited 5.3-fold and 2.5-fold increases in expression in root samples of Seosan-inoculated plants at day 3 and day 14, respectively, compared to mock-treated plants (Figure 4).

### 2.4. Expression Profiles of BrIAA and BrRACK Genes

**Figure 5:**
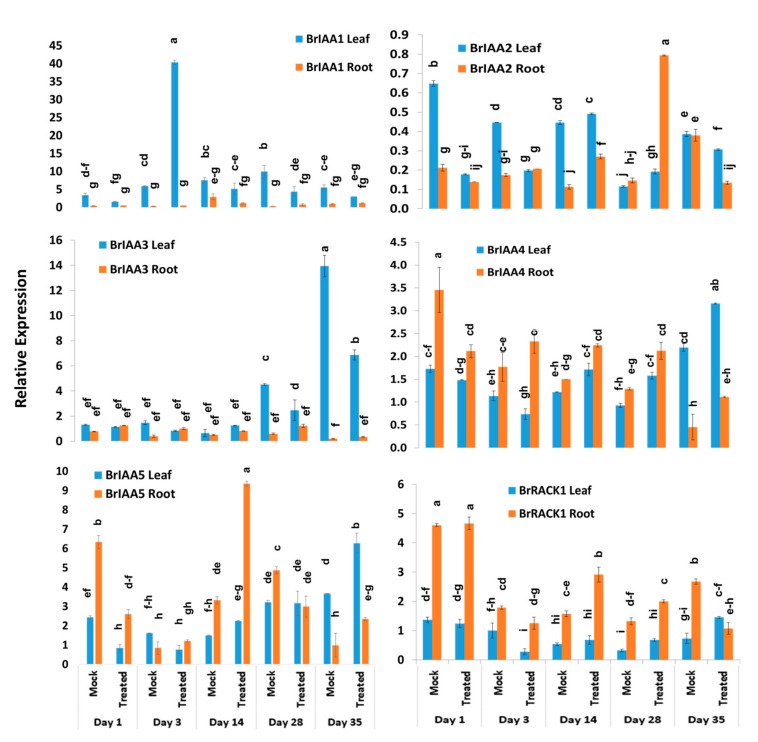
Variation in relative expression levels of auxin-related genes *BrIAA* and *BrRACK1* genes in leaf and root samples under different treatment × time point combinations: Data represent relative expression levels of three different treatment types—sample type (leaf vs. root), treatment (Seosan-inoculated vs. mock-treated), and time-points. Vertical bars represent standard deviation. Each data point estimates the average of three independent biological replicates. Data bars separated by different letters indicate 5% statistical significance level according to Tukey’s pairwise comparisons. Expression of genes in leaf tissues of control (nontreated) plants on day 0 were set to 1.

*BrIAA1–BrIAA5* genes exhibited significant variations between five time-points, treatments, treatment × time-points, and sample types within treatment and time-point (Table 2). The *BrIAA1* gene showed significantly higher expression in leaf samples compared to root samples (Figure 5). This gene showed 6.9-fold increase in expression at day 3 in leaf samples of Seosan-inoculated plants compared to mock-treated leaf samples (Figure 4). The *BrIAA2* gene showed the highest expression level in root tissues at day 28 in Seosan-inoculated plants. This gene exhibited 5.4-fold increase in expression in Seosan-inoculated root tissues compared to mock-treated root tissues at day 28 (Figure 4 and Figure 5). The *BrIAA3* gene showed the highest expression level in mock-treated leaf tissues on day 35, and the *BrIAA4* gene showed the highest expression in mock-treated leaf tissues at day 1 (Figure 5). The *BrIAA5* gene showed the highest expression in Seosan-inoculated root tissues on day 14. This gene upregulated 2.8-fold in Seosan-inoculated root tissues compared to mock-treated root tissues at day 14 (Figure 4 and Figure 5). In addition, the *BrRACK1* gene exhibited comparatively higher relative expression in root tissues compared to leaf tissues (Figure 5).

### 2.5. Expression Profiles of BrSAUR and BrNIT Signaling Genes

**Figure 6:**
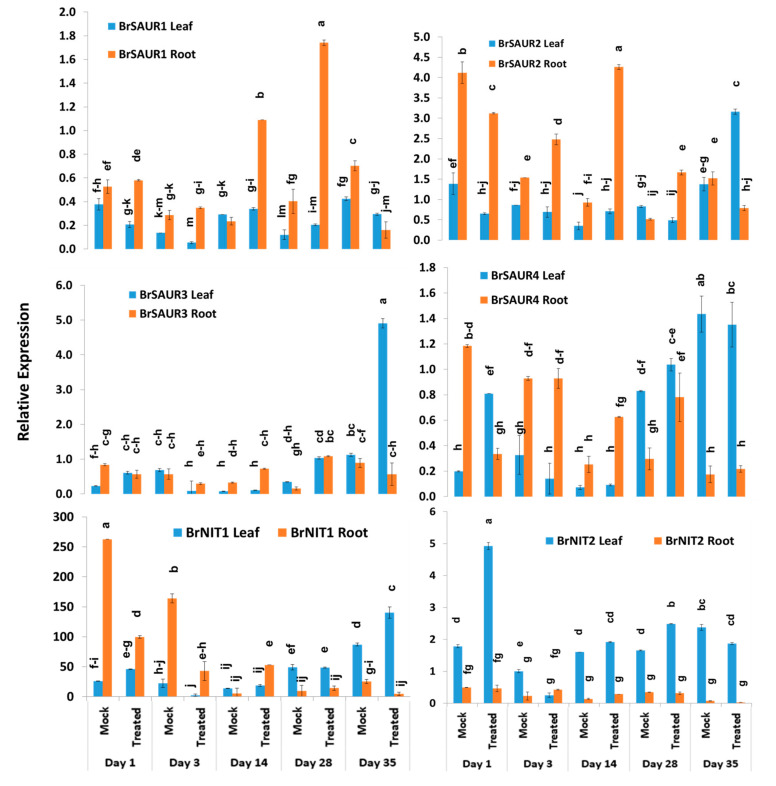
Variation in the relative expression levels of auxin-related genes *BrNIT* and *BrSAUR* genes in leaf and root samples under different treatment × time-point combinations: Data represent relative expression levels of three different treatment types—sample type (leaf vs. root), treatment (Seosan-inoculated vs. mock-treated), and time-points. Vertical bars represent standard deviation. Each data point estimates the average of three independent biological replicates. Data bars separated by different letters indicate the 5% statistical significance level according to Tukey’s pairwise comparisons. Expression of genes in leaf tissues of control (nontreated) plants on day 0 were set to 1.

The expression levels of four *SAUR*-like auxin-responsive genes varied significantly between time-points, treatment × time-points, and sample types within treatment and time-point (Table 2). The *BrSAUR1* gene exhibited the highest expression level in root tissues of Seosan-inoculated plants on day 28 followed by day 14 (Figure 6). This gene upregulated 4.6- and 4.3-fold in root tissues of Seosan-inoculated plants on day 14 and day 28, respectively, compared to mock-treated root tissues (Figure 4 and Figure 6). The *BrSAUR2* gene showed higher expression levels in root tissues of Seosan-inoculated plants on day 14 and mock-treated root tissues on day 1 compared to other samples (Figure 6). The *BrSAUR3* gene exhibited 4.3-fold increase in expression on day 35 in leaf tissues of Seosan-inoculated plants compared to mock-treated plants (Figure 4 and Figure 6). The *BrSAUR4* gene showed higher expression in root tissues on day 1 and day 3 but lower expression on day 28 and day 35 compared to leaf tissues (Figure 6). The nitrilase gene, *BrNIT1*, showed the highest expression level in mock-treated root tissues on day 1, and the expression of this gene reduced in root tissues with progression of clubroot formation (Figure 6). Another nitrilase gene, *BrNIT2*, showed upregulation in relative expression in leaf tissues compared to root tissues. This gene exhibited 4.6- and 3.2-fold increases in expression in root tissues of Seosan-inoculated plants compared to mock-treated plants on day 14 and day 28, respectively (Figure 4 and Figure 6).

### 2.6. Expression Profiles of Other Auxin-Related Genes

**Figure 7:**
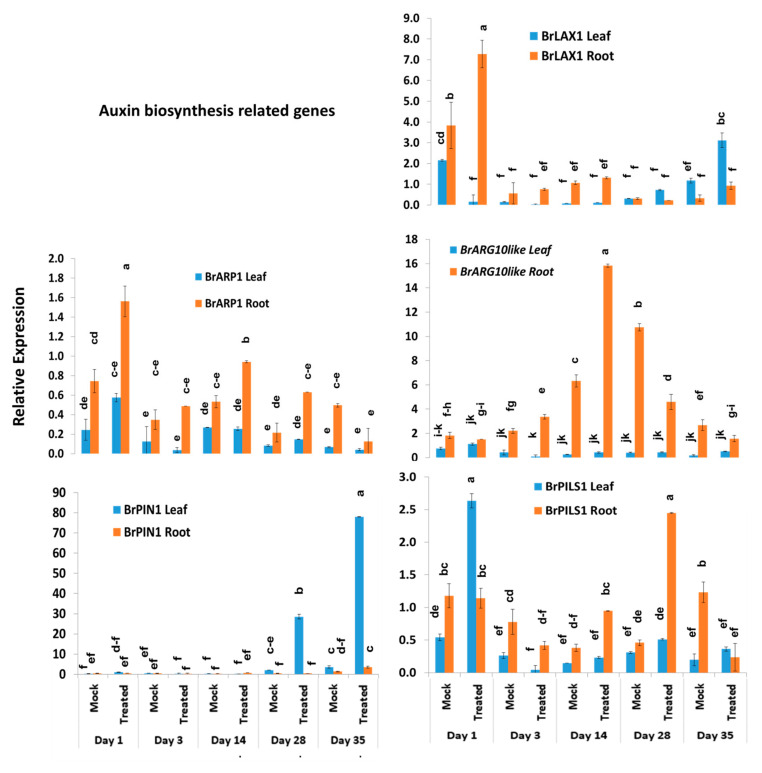
Variation in the relative expression levels of other auxin-related genes in leaf and root samples under different treatment × time-point combinations: Data represent relative expression levels of three different treatment types—sample type (leaf vs. root), treatment (Seosan-inoculated vs. mock-treated), and time-points. Vertical bars represent standard deviation. Each data point estimates the average of three independent biological replicates. Data bars separated by different letters indicate the 5% statistical significance level according to Tukey’s pairwise comparisons. Expression of genes in leaf tissues of control (nontreated) plants on day 0 were set to 1.

Among the other five auxin biosynthesis-related genes, the *BrLAX1* gene exhibited 1.9-fold increase in expression in root tissues of Seosan-inoculated plants compared to mock-treated plants on day 1 (Figure 4 and Figure 7). The auxin-repressed protein synthesizing gene, *BrARP1*, upregulated 2.1-, 1.8-, and 2.9-fold in root tissues of Seosan-inoculated plants compared to mock-treated plants on day 1, day 14, and day 28, respectively (Figure 4 and Figure 7). The *BrARG10like* gene exhibited 2.5-fold increase in expression in root tissues Seosan-inoculated plants compared to mock-treated plants on day 14 (Figure 4 and Figure 7). Relative expression of *BrARG10like* gene was notably higher in root tissues compared leaf tissues at all time-points (Figure 7). The *BrPIN1* gene expressed highly in leaf tissues compared to root tissues, in general (Figure 7). This gene showed 13.7- and 21.9-fold increases in expression in leaf tissues of Seosan-inoculated plants compared to mock-treated plants on day 28 and day 35, respectively (Figure 4 and Figure 7). The *BrPILS1* gene showed 4.9-fold increase in expression in leaf tissues on day 1 and 5.3-fold increase in expression in root tissues on day 28 in Seosan-inoculated plants compared to mock-treated plants (Figure 4 and Figure 7).

## 3. Discussion

The major focus of the study was to investigate the transcript profiles of 23 auxin-related genes at both the primary and secondary phases of clubroot infection in Chinese cabbage after inoculation with *P. brassicae*. Auxins are the phytohormones that regulate a number of physiological and biochemical processes in roots and shoots during clubroot disease development [1,22,26]. In this study, we used a highly virulent isolate of *P. brassicae* to inoculate the Chinese cabbage line to obtain a remarked alteration in transcript levels of auxin-related genes [29].

### 3.1. Expression Level Difference in Leaf vs. Root Tissue

Like cytokinins, auxins also play a vital role in root–shoot homeostasis [30,31]. These phytohormones produced in both shoots and root tips particularly promote elongation of stem and inhibit proliferation of lateral buds other than their role in plant–microbe interactions [32,33,34]. Several auxin-related genes in this study dynamically changed in leaf and root tissues of both mock-treated and Seosan-inoculated samples at both the primary and secondary phases of infection, indicating that auxin homeostasis at the intra-plant level is dynamic and that the auxin level alters over time between roots and shoots (Figure 3, Figure 4, Figure 5, Figure 6 and Figure 7). The *BrGH3.3, BrDCK1*, and *BrARG10like* genes were comparatively highly expressed by 84.4-, 28.2-, and 10.9-fold, respectively, in root tissues compared to leaf tissues, indicating that these three genes are important in auxin-mediated root development (Figure 2). In contrast, four genes, *BrGH3.2, BrIAA1, BrPIN1*, and *BrNIT2*, were highly expressed in leaf tissues compared to root tissues, indicating that these genes have a vital role in auxin-mediated leaf development (Figure 2). The variation in expression levels of auxin-related genes in leaves and roots further indicated that the auxin level differs between roots and leaves considering that expression level of auxin-signaling, receptor, and transporter genes are positively associated with auxin biosynthesis and accumulation in different organs.

### 3.2. Expression Level Difference and Role of Auxin Signaling and Biosynthesis (BrGH3, BrIAA, BrNIT, and BrSAUR) Genes

**Figure 8:**
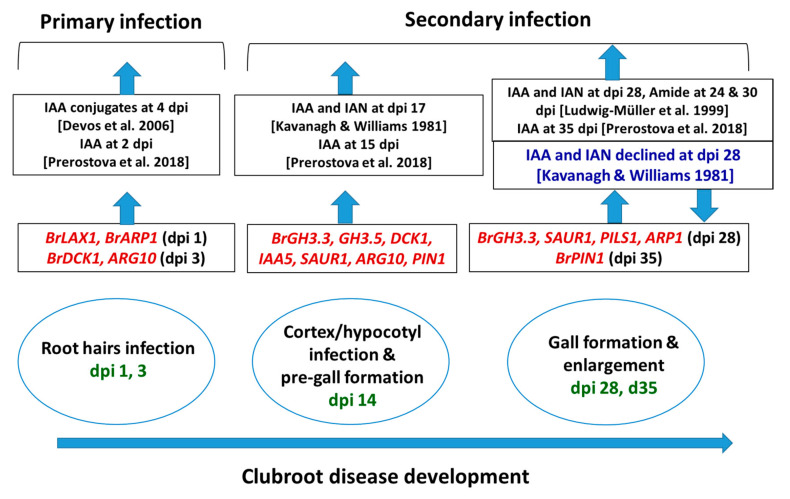
Relative between expression levels of auxin signaling, receptor, transporter, and repressed genes in this study (red color) and auxin contents in the published reports at the adjacent time-points, in *Arabidopsis thaliana* and *Brassica spp*. dpi, days post inoculation: Green color represents the dpi at which samples were collected. Upwards- and downwards-pointing arrows represent increase and decrease (blue text) in transcript abundance of genes or auxin accumulation, respectively, in clubroot-infected plants compared to non-inoculated plants.

*BrGH3*, *BrIAA*, *BrNIT*, and *BrSAUR* are the primary responsive genes that play a vital role in auxin signaling and biosynthesis [35,36]. In *A. thaliana*, several members of *AtGH3*, auxin conjugate synthetases (Gretchenhagen-3), including *GH3.2*, *GH3.3*, *GH3.4*, *GH3.5*, *GH3.14*, and *GH3.17* were upregulated at 24 and 28 dai in the *P. brassicae*-inoculated plants compared to non-inoculated plants [19]. Among them, the *AtGH3.5* gene plays multiple roles in auxin biosynthesis by conjugating with IAA and salicylic acid, and this gene has a role in camalexin biosynthesis [19]. GH3 proteins by conjugating with IAA possibly play a role in auxin homeostasis and plant–pathogen interactions [19,37]. In this study, a remarkable upregulation of the *BrGH3.3* gene at 14 and 28 dai and the *BrGH3.5* gene at 14 dai in the root tissues of Seosan-inoculated plants compared to mock-treated plants indicates that these two genes have a vital importance in clubroot formation through enhanced accumulation of auxins or auxin conjugating enzymes (Figure 8) [19].

Experimental evidence indicated that IAA transport has a role in clubroot formation [3,24]. A few *IAA* signaling genes such as *AtIAA7* [19] and *AtIAA2* [24] were upregulated in previous studies in the infected root tissues but the transcript abundance of *AtIAA28* was higher in control plants compared to infected plants [19]. *AtIAA28* was also found to negatively regulate lateral root formation [38], where hyperplasia and massive lateral root formation occurred simultaneously during clubroot formation [19] indicating that upregulation of *AtIAA28* may inhibit clubroot formation. In this study, none among the five *BrIAA* genes were markedly upregulated in root tissues (Figure 2) except that the *BrIAA5* gene was 2.8-fold upregulated at 14 days after inoculation in treated plants, indicating that this gene might play role in auxin signaling during cortex infection (Figure 4 and Figure 8).

Two genes *BrNIT1* and *BrNIT2* were from the nitrilases protein family that are associated with the biosynthesis of IAA. Nitrilases are involved in catalyzing the conversion of IAN (indole-3-acetonitrile) to IAA in root tissues of *A. thaliana* [39,40,41]. Both nitrilase 1 and 2 were actively involved in clubroot formation [17,23]. Transformation of *A. thaliana* plants with nitrilase 1 with antisense direction (aNIT1) exhibited a reduced infection rate cogmpared to wild type plants [17]. In this study, a 4.6-fold higher expression of *BrNIT1* gene in the Seosan-inoculated root tissues at 14 days after inoculation compared to mock-treated root tissues indicated that nitrilase enzyme might be activated for auxin signaling during cortex infection (Figure 4). Our assumption is consistent with a previous study that observed a strikingly higher expression of *NIT1* gene in susceptible cultivar of *B. napus*–Hornet compared to the comparatively resistant cultivar Alister [42].

Another group of auxin signaling genes was *SAUR*-like genes which are transiently expressed in response to auxin. The *SAUR*-genes, early auxin-responsive gene family in *A. thaliana* are involved in plant development and leaf senescence [43,44,45]. In *A. thaliana*, *SAUR* genes were highly upregulated in the root tissues at 17 dpi after *P. brassicae* inoculation, indicating that IAA induces root cell division during pre-gall formation and cortex infection [46]. In contrast, Ciaghi et al. [47] reported upregulation of *SAUR* genes in symptomless root tissues. In this study, an increase in expression of *BrSAUR1* gene by 4.6- and 4.3-folds in Seosan-inoculated plants compared to mock-treated plants at 14 and 28 after inoculation, respectively, indicated that this gene has important role in auxin signaling during gall formation (Figure 4 and Figure 8).

### 3.3. Expression Level Difference and Role of Auxin Transporter (BrPIN, BrPILS, and BrLAX) Genes

PIN, PILS, and LAX proteins are involved in mediating the transport of auxin across the plasma membrane. The subcellular localization of PIN, auxin efflux carriers, largely determine the direction of auxin transport. Subcellular dynamics of PIN proteins is vital for the directionality of auxin transport [48,49]. In addition, distribution of auxin, collectively regulated by five *PIN* genes in the primary roots of *Arabidopsis*, regulates cell division and cell expansion [50]. In this study, 3.7- and 2.7-fold upregulation of the *BrPIN1* gene at 14 and 35 days after inoculation, respectively, and 5.3-fold upregulation of the *BrPILS1* (PIN-like auxin efflux carrier) gene at 28 days after inoculation in Seosan-inoculated roots compared to mock-treated samples indicated that these two genes have vital importance in auxin distribution in dividing cells during cortex infection and gall formation (Figure 4 and Figure 8). AUX/LAX family members and auxin influx transporters are involved in root gravitropism and root hair development along with other activities [51]. In this study, a higher expression of *BrLAX1* gene in Seosan-inoculated roots by 1.9-fold at 1 dpi indicated that this gene played a positive role in root hair infection during the primary infection phase of *P. brassicae* (Figure 4 and Figure 8).

### 3.4. Expression Level Difference and Role of Auxin Receptor (BrDCK and BrRACK) Genes

*BrDCK1* and *BrRACK1* are the auxin receptor genes that were not studied before during clubroot disease development. However, experimental data suggested that *RACK1* gene, by interacting with signaling molecules and by playing its multiple hormone responsiveness role, may modulate signal transduction pathways [52,53]. Overexpression of *BoRACK1* gene in kale (*B. oleracea* var. acephala) resulted in the reduction of downy mildew symptoms caused by *Peronospora brassicae,* indicating that this gene may develop a network with regulatory genes to offer resistance to downy mildew disease [53]. In contrast, BjuTIR1/AFB family genes are the auxin receptor genes that were found to be greatly induced by *P. brassicae* treatment, indicating that upregulation of auxin receptor genes may positively regulate clubroot formation [54]. In this study, induced expression levels of *BrDCK1* gene by 5.3- and 2.5-fold in Seosan-inoculated plants compared to mock-treated plants at 3 dpi and 14 dpi, respectively, indicate that this gene has importance in both primary infection and cortex infection (Figure 4 and Figure 8).

### 3.5. Expression Level Difference and Role of BrARP1 Gene

Auxin-repressed proteins (ARPs) are responsive to various biotic and abiotic stress factors including drought [55,56], salinity [56,57], cold/chilling [56,57,58], heat [57], insect [59], and *S. sclerotiorum* fungus [59]. In *Arabidopsis*, the *BnARP1-OE-135* transgenic line exhibited 70% seedling survival whereas the wild type plants showed less than 20% seedling survival upon the inoculation of *S. sclerotiorum*, indicating that a higher expression level of the *BnARP1* gene was associated with seedling resistance [59]. Overexpression of the *BrARP1* gene in *Arabidopsis* reduced vegetative growth and seed productivity, indicating that this gene is involved in growth arrest [57]. A notably higher expression of the *BrARP1* gene by 2.1-, 1.8-, and 2.9-fold at 1, 14, and 28 dpi, respectively, in the Seosan-inoculated root tissues compared to mock-treated roots indicated that this gene remains active at both the primary and secondary phases of infection to tackle *P. brassicae* infection. However, the development of disease is a subject of interactions among many other phytohormonal cross-talks including auxins, cytokinins, brassinosteroids, salicylic acid, etc. [29,42]. Contents of IAA and IAN accumulated differentially in *B. oleracea* (cabbage) and *Arabidopsis* at 28 dpi in previous studies (Figure 8). Ludwig-Müller et al. [7] observed an increase in accumulation of IAA and IAN in clubroots of *A.* thaliana whereas Kavanagh and Williams [60] observed a decrease in contents of these two auxins in cabbage at 28 dpi. Content of amide was also increased at both 24 and 30 dpi in *A. thaliana* [7]. We predict that a higher expression of auxin repressed genes (*ARP1*) might be one of the factors of decreasing in IAA and IAN in the clubroots of cabbage (Figure 8).

## 4. Materials and Methods

### 4.1. Preparation of Plant Materials

The Chinese cabbage (*Brassica rapa* var. pekinensis) cultivar “Bullam-3-ho” that was collected from Woori Seeds company, South Korea, was selected for inoculation with *P. brassicae.* Collected seeds were sterilized in 70% ethanol for 1 min and then in 1% sodium hypochlorite (NaOCl) for 8 min and washed by double distilled water (DDW) for three times in turn. For germination and seedling establishment, the seeds were then planted into 50-cell trays (tray size: 540 mm × 280 mm; hole size: 46 mm × 23 mm × 45 mm) containing sterile nursery soil mixture. Plants were grown under controlled conditions at 25 ± 1 °C temperature, 65–70% relative humidity, and 230–250 μmol m^-2^ s^-1^ light intensity for a 16:8 h light-dark cycle.

### 4.2. Gall Collection, Spore Preparation, and Inoculation of Plant Materials

**Figure 9:**
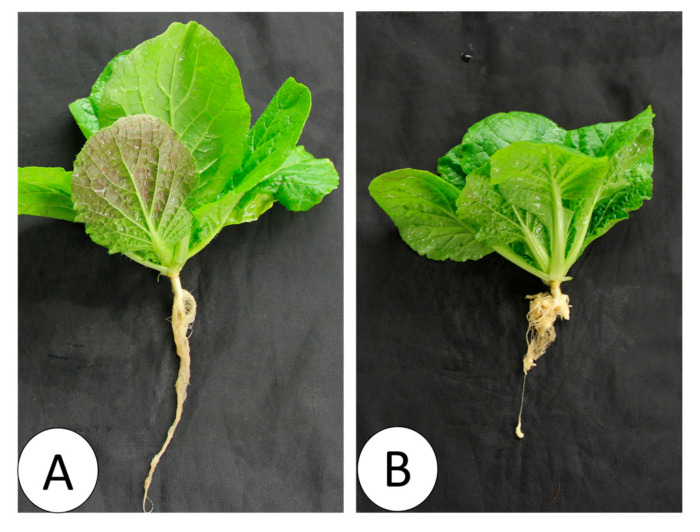
Nontreated (**A**) and Seosan-inoculated plants (**B**) of Chinese cabbage cultivar “Bullam-3-ho” at 35 days after inoculation of the “Seosan” isolate of *P. brassicae*.

*P. brassicae* spores of the “Seosan” isolate, a highly virulent isolate, were extracted according to Feng et al. [61] and Laila et al. [29]. Extracted spores were counted in a hemocytometer, and the final concentration was adjusted to 1 × 10^7^ spores µL^−1^. For the disease inoculation, we followed the root cutting method. Two-week-old seedlings were inoculated with prepared spores of the Seosan isolate of *P. brassicae*, and the inoculated plants were monitored for the following five weeks for any gall formation (Figure 9). After cutting the root branches, plants were inoculated with 6 mL of spore suspension (10^7^ spores µL^-1^) for 10–15 minutes, and to maintain a stable growth environment, the inoculated plants were immediately transferred in a 200 cm^3^ pot containing artificial soil in a controlled growth chamber. The roots of plants under the mock treatment were incubated in 6 mL sterile water.

### 4.3. Sampling of Inoculated Roots and Leaves for Expression Analysis

For expression study, the Chinese cabbage cultivar “Bullam-3-ho” was inoculated with the *P. brassicae* isolate “Seosan” (pathotype 4) at 10^7^ spores mL^-1^. This isolate was found to be the most virulent compared to three other Korean geographical isolates of *P. brassicae* in a recent study [29]. For the qRT-PCR expression analysis of selected auxin-related genes, roots and leaves were sampled from 14-day-old mock and inoculated plants at 1, 3, 14, 28, and 35 dai as listed in Table 3. Leaf samples from nontreated plants were collected on day 0 as a reference. All the sampling was done in triplicate. After uprooting, the plant roots were washed carefully under running tap water to remove soil particles, followed by drying the roots keeping on filter papers. The samples were then immediately frozen in liquid nitrogen and stored at −80 °C prior to use.

### 4.4. Extraction of RNA

For extracting total RNA, RNeasy Plant Mini Kit (Qiagen, Hilden, Germany) was used. To extract the total RNA from all 20 samples (Table 3), about 100 mg of leaf or root tissue was taken into a mortar. Samples were homogenized with a pestle in liquid nitrogen. Traces of DNA were eliminated using RNase-free DNase (Qiagen). The level of purity of the isolated RNA was measured at a 260/280 nm ratio in the NanoDrop^®^ ND-1000 (Thermo Scientific, Hudson, NH, USA). Total RNA was converted to complementary DNA (cDNA) using a Superscript^®^ III First-Strand Synthesis Kit (Invitrogen, CA, USA) following the manufacturer’s instructions.

### 4.5. In Silico Analysis of Auxin-Related Genes

The list of auxin metabolism, signaling, and transport-related genes in *A. thaliana* reported by Schuller et al. [21] was used as baseline to find corresponding auxin-related *B. rapa* orthologs from the BRAD database (http://brassicadb.org/brad/; Cheng et al. [62]; Table 1, Figure S1). In silico analysis was performed to assess the gene properties. For analyzing the primary structures of the genes, protParam (http://expasy.org/tools/protparam.html) [63] was used. Localization of the auxin biosynthetic proteins was predicted using both DeepLoc-1.0 (http://www.cbs.dtu.dk/services/DeepLoc/) and UniProt (https://www.uniprot.org/uniprot/). A phylogenetic tree was generated using Neighbor-joining (NJ) algorithm [64] in MEGA X software [65].

### 4.6. Quantitative RT-PCR for Expression Analysis

The relative expression patterns of *B. rapa* auxin-related genes were studied using quantitative RT-PCR (qPCR) in 20 samples presented in Table 3. Expression of genes in leaf tissues of control (nontreated) plants on day 0 were set to 1. Each of three biological replicates were repeated three times. The gene-specific primers designed by Primer3 software (http://frodo.wi.mit.edu/primer3) were used for qPCR expression analysis (Table 4). Primer specificity was tested by following Robin et al. [66]. GenBank Accession Nos. XM_009147610.2 and FJ969844.1 from *B. rapa* representing ACTIN genes (housekeeping genes) were used to standardize the expression levels of 23 auxin-related genes. The qPCR reaction was performed using 1 μL cDNA template of 50 ng μL^-1^ concentration, 1 μL each of forward and reverse primers at 10 pmol concentration, 10 μL qPCR BIOSyGreen Mix Lo-ROX (PCR Biosystems, London, UK), and 7 μL double distilled water to make a final reaction volume of 20 μL. The conditions for qPCR were as follows: denaturation for 10 min at 95 °C, followed by 40 cycles of amplification at 95 °C for 20 s, 58 °C for 20 s, and 72 °C for 25 s. The fluorescence was recorded following the last step of each cycle for three technical replicates per sample. For the detection of amplification and analyzing data, LightCycler96 (Roche, Mannheim, Germany) software was used. The relative expression value of nontreated leaf samples at day 0 was set to 1. The relative transcript abundance of the auxin-related genes was calculated using the 2^−ΔΔ*C*t^ method [67].

### 4.7. Statistical Analysis

A nested analysis of variance (ANOVA) under general linear model was employed to depict significant difference in relative expression levels of auxin biosynthesis-related genes for treatments (mock and Seosan-inoculated), time-points (dpi), and sample types (leaf vs. root) using MINITAB 17 Statistical Software (Minitab Inc., State College, PA, USA). A one-way ANOVA was carried out to explore the statistical significance between leaf vs. root using MINITAB 17 Statistical Software. Tukey’s pairwise comparisons were used for comparing means of relative expression values.

## 5. Conclusions

Expression levels of auxin signaling, transport, receptor, and repressed-related genes showed notable variation between leaf and root tissues, indicating their organ-specific differential expression. The *BrGH3.3, BrDCK1, BrNIT1*, and *BrARG10like* genes exhibited remarkably higher expression in root tissues compared to leaf tissues, indicating their importance in clubroot development. The *BrGH3.3, BrNIT1, BrPIN1*, and *BrDCK1* genes were markedly induced in *P. brassicae*-infected root tissues at 14 dpi during cortex infection and played a role in auxin signaling that is required for clubroot formation. The *BrGH3.3, BrPIN1*, and *BrPILS1* genes were induced at 28 dpi and played a role in auxin accumulation for cell division and expansion during gall formation. *BrLAX1* was induced at 1 dpi that possibly enhanced primary infection at root hairs. In contrast, a notably higher expression of *BrARP1* gene in infected root tissues at three different dpi indicated resistance response of host plants towards clubroot development. We conclude that the *BrGH3.3, BrNIT1, BrPIN1*, and *BrDCK1* genes play a significant role in clubroot formation via auxin signaling. Developing mutants of these four genes, coupled with a higher expression of *BrARP1* gene, may offer clubroot resistance in Chinese cabbage.

## Figures and Tables

**Table 1:** Properties of genes involved in auxin metabolism, signaling, and transport in *Brassica rapa.* St., strand; aa, amino acid; Pi, isoelectric point, MW, molecular weight. ‘+’ indicates 5’-3’ direction and ‘-‘ indicates 3’-5’ direction of DNA.

Gene Name	BRAD ID	Chromosomal Location	Start Codon	Stop Codon	St.	Gene Identity	(aa)	Pi	MW (kDa)	Subcellular Localization
*BrDCK1*	Bra008038	A02	11763540	11765539	−	Receptor kinase	447	9.06	50.44	Cell membrane
*BrDCK1.1*	Bra016050	A07	22960115	22961810	−	Protein kinase	451	9.24	50.77	Cell membrane
*BrDCK1.2*	Bra003875	A07	18779483	18780917	+		396	8.74	44.61	Cell membrane
*BrLAX1*	Bra008325	A02	13732347	13734803	−	Auxin efflux carrier/ Transporter	472	8.95	53.53	Cell membrane
*BrLAX1.1*	Bra003674	A07	17668825	17671143	+		471	8.86	53.45	Cell membrane
*BrPIN1*	Bra015983	A07	23234264	23237329	+		618	9.14	66.82	Cell membrane
*BrPIN1.1*	Bra008105	A02	12154076	12156932	+		616	9.10	66.61	Cell membrane
*BrPILS1*	Bra014265	A08	2101045	2103020	−		431	9.26	46.66	Lysosome
*BrPILS1.1*	Bra030441	A05	11329601	11331470	+		423	8.86	46.08	Cell membrane
*BrRACK1*	Bra032261	A05	12304734	12306049	+	Receptor C kinase	327	6.31	35.70	Nucleus
*BrRACK1.1*	Bra018745	A06	2234607	2235718	+		298	6.06	32.47	Nucleus
*BrRACK1.2*	Bra022282	A05	19371512	19372720	+		325	7.59	35.46	Nucleus
*BrRACK1.3*	Bra021294	A01	21899789	21900926	+		326	8.33	35.58	Nucleus
*BrGH3.1*	Bra039186	A09	33153634	33155891	+	Auxin-responsive *GH3* (auxin signaling)	596	6.28	67.77	Cytoplasm
*BrGH3.2*	Bra008833	A10	12836737	12839205	−		594	7.81	67.08	Cytoplasm
*BrGH3.3*	Bra041046	Scaffold000414	2738	4832	−		587	5.98	66.03	Cytoplasm
*BrGH3.31*	Bra023405	A02	2218215	2221058	+		595	5.80	67.02	Cytoplasm
*BrGH3.4*	Bra034205	A09	1271960	1274617	−		610	6.28	68.54	Cytoplasm
*BrGH3.41*	Bra013086	A03	20567114	20569305	−		557	5.43	62.48	Cytoplasm
*BrGH3.5*	Bra019060	A03	26541024	26543324	+		612	5.98	69.39	Cytoplasm
*BrGH3.51*	Bra026365	A01	9560154	9562651	+		612	6.05	69.36	Cytoplasm
*BrGH3.52*	Bra022725	A02	6752640	6754845	+		612	5.87	69.09	Cytoplasm
*BrGH3.53*	Bra029023	A03	5931112	5933213	+		608	5.74	68.63	Cytoplasm
*BrIAA1*	Bra039855	A08	6303677	6305559	−	Auxin signaling	233	8.68	25.85	Nucleus
*BrIAA2*	Bra033886	A05	15764615	15766543	−		242	8.78	26.43	Nucleus
*BrIAA2.1*	Bra001900	A03	19263301	19265148	+		237	8.54	26.16	Nucleus
*BrIAA3*	Bra001634	A03	17624668	17625966	−	Phytochrome -associated	272	7.68	30.43	Nucleus
*BrIAA3.1*	Bra021184	A01	22676134	22677514	+		269	9.20	30.28	Nucleus
*BrIAA4*	Bra009867	A06	18399781	18400911	+	Auxin signaling	176	8.80	20.33	Nucleus
*BrIAA4.1*	Bra036557	A09	2815416	2816543	−		175	9.24	20.00	Cytoplasm
*BrIAA5*	Bra032521	A09	38191234	38191967	+	Transcriptional regulator	180	5.11	20.36	Cytoplasm
*BrIAA5.1*	Bra015297	A10	2137104	2137826	+		186	5.31	21.10	Cytoplasm
*BrSAUR1*	Bra011560	A01	1680948	1681271	+	Auxin-responsive protein (auxin signaling)	107	6.82	12.11	Mitochondrion
*BrSAUR1.1*	Bra034651	A08	11488514	11488828	+		104	8.60	12.03	Mitochondrion
*BrSAUR1.2*	Bra017676	A03	29740606	29740929	−		107	6.82	12.14	Mitochondrion
*BrSAUR1.3*	Bra013061	A03	20715121	20715447	−		108	7.73	12.36	Mitochondrion
*BrSAUR2*	Bra039345	Scaffold000164	28744	29145	−		133	9.03	15.34	Mitochondrion
*BrSAUR3*	Bra039264	A04	18962112	18962471	+		119	7.81	13.67	Cell membrane
*BrSAUR3.1*	Bra004515	A05	560573	560941	−		122	8.79	14.06	Cell membrane
*BrSAUR4*	Bra014476	A04	882244	882756	−		170	10.96	19.75	Mitochondrion
*BrNIT1*	Bra028932	A03	5467727	5469454	−	Auxin biosynthesis	343	5.54	37.73	Cytoplasm
*BrNIT1.1*	Bra021681	A04	13934901	13936735	+		350	5.48	38.48	Cytoplasm
*BrNIT2*	Bra020184	A02	4417472	4419242	+		357	5.30	38.94	Cytoplasm
*BrARG10-like*	Bra026348	A01	9659581	9660796	−	Auxin responsive	250	5.52	27.55	Cytoplasm
*BrARG10-like1*	Bra010404	A08	13579603	13580693	+		250	6.07	27.49	Cytoplasm
*BrARP1*	Bra013191	A03	19982046	19983742	+	Auxin repressed protein	403	9.70	43.86	Cell membrane

**Table 2:** Analysis of variance for relative expression levels of 23 auxin-biosynthesis related genes in two different sample types (leaf vs. root—LvR) at five different time-points (Tm) (1, 3, 14, 28, and 35 days after inoculation) under two different treatments (Tr, mock-treatment vs. inoculation with Seosan-isolate of *P. brassicae*) in Chinese cabbage cultivar “Bullam-3-ho”.

Sources of Variation	df	Genes	MS	F Statistic	*p* Value	Genes	MS	F Statistic	*p* Value	Genes	MS	F Statistic	*p* Value
Time-point	4	*BrGH3.1*	2.68	83.2	< 0.01	*BrIAA4*	1.01	63.4	< 0.01	*BrDCK1*	132.7	76.3	< 0.01
Treatment	1		0.94	29.2	< 0.01		1.28	80.2	< 0.01		22.1	12.7	< 0.01
Tm × Tr	4		1.34	41.8	< 0.01		1.35	84.5	< 0.01		28.5	16.4	< 0.01
LvR (Tm Tr)	10		1.21	37.7	< 0.01		2.14	134.3	< 0.01		265	152.9	< 0.01
Error	40		0.032				0.016				1.74		
Time-point	4	*BrGH3.2*	1519	19.8	< 0.01	*BrIAA5*	15.5	71.7	< 0.01	*BrRACK1*	11.67	69.7	< 0.01
Treatment	1		2169	28.2	< 0.01		1.14	5.28	0.027		0.116	0.70	0.409
Tm × Tr	4		1184	15.4	< 0.01		16.7	77.3	< 0.01		1.11	6.66	< 0.01
LvR (Tm Tr)	10		1408	18.3	< 0.01		14.2	65.9	< 0.01		8.06	48.2	< 0.01
Error	40		76.8				0.22				0.167		
Time-point	4	*BrGH3.3*	4,699,114	2767	< 0.01	*BrSAUR1*	0.268	406.6	< 0.01	*BrLAX1*	14.2	128.2	< 0.01
Treatment	1		1,822,177	1073	< 0.01		0.345	522.1	< 0.01		3.52	31.7	< 0.01
Tm × Tr	4		4,623,051	2723	< 0.01		0.533	805.8	< 0.01		0.80	7.24	< 0.01
LvR (Tm Tr)	10		6,320,827	3723	< 0.01		0.505	765.3	< 0.01		9.12	82.2	< 0.01
Error	40		1698				0.0007				0.11		
Time-point	4	*BrGH3.4*	1978.9	582.8	< 0.01	*BrSAUR2*	3.56	46.24	< 0.01	*BrARP1*	0.726	158	< 0.01
Treatment	1		54.83	17.0	< 0.01		3.63	47.22	< 0.01		0.422	91.8	< 0.01
Tm × Tr	4		161.8	50.2	< 0.01		2.93	38.03	< 0.01		0.243	52.9	< 0.01
LvR (Tm Tr)	10		1369.7	424.8	< 0.01		5.61	72.92	< 0.01		0.368	80.1	< 0.01
Error	40		3.22				0.077				0.004		
Time-point	4	*BrGH3.5*	396.0	72.82	< 0.01	*BrSAUR3*	4.84	355.6	< 0.01	*BrARG*	52.95	741.5	< 0.01
Treatment	1		71.8	13.21	< 0.01		3.37	247.5	< 0.01		2.62	36.6	< 0.01
Tm × Tr	4		215.8	39.69	< 0.01		2.06	151.0	< 0.01		26.39	369.6	< 0.01
LvR (Tm Tr)	10		357.9	65.82	< 0.01		2.96	218.0	< 0.01		65.54	917.8	< 0.01
Error	40		5.44				0.013				0.071		
Time-point	4	*BrIAA1*	200.9	192.5	< 0.01	*BrSAUR4*	0.518	121.1	< 0.01	*BrPIN1*	260.1	13.2	< 0.01
Treatment	1		76.18	73.0	< 0.01		0.057	13.44	< 0.01		298.8	15.1	< 0.01
Tm × Tr	4		213.8	204.8	< 0.01		0.121	28.35	< 0.01		168.5	8.54	< 0.01
LvR (Tm Tr)	10		266.2	255.1	< 0.01		0.857	200.5	< 0.01		206.0	10.4	< 0.01
Error	40		1.04				0.004				19.7		
Time-point	4	*BrIAA2*	0.017	12.15	< 0.01	*BrNIT1*	14844	165.8	< 0.01	*BrPILS1*	0.969	233.9	< 0.01
Treatment	1		0.013	9.57	< 0.01		9378	104.8	< 0.01		0.789	190.5	< 0.01
Tm × Tr	4		0.173	120.9	< 0.01		6064	67.7	< 0.01		0.695	167.6	< 0.01
LvR (Tm Tr)	10		0.129	90.6	< 0.01		15511	173.3	< 0.01		0.584	140.9	< 0.01
Error	40		0.0014				89.5				0.004		
Time-point	4	*BrIAA3*	47.3	632.4	< 0.01	*BrNIT2*	1.55	22.95	< 0.01				
Treatment	1		8.28	110.7	< 0.01		0.85	12.67	< 0.01				
Tm × Tr	4		8.16	109.2	< 0.01		1.05	15.52	< 0.01				
LvR (Tm Tr)	10		40.1	535.5	< 0.01		2.78	41.08	< 0.01				
Error	40		0.075				0.067						

**Table 3:** Leaf and root samples collected from Seosan-inoculated and mock-treated plants at five different time points under two different treatments.

Sample ID	Time Point	Treatment	Sample Type
1	Day 1	Mock	Leaf
2	Day 1	Mock	Root
3	Day 1	Seosan-inoculated	Leaf
4	Day 1	Seosan-inoculated	Root
5	Day 3	Mock	Leaf
6	Day 3	Mock	Root
7	Day 3	Seosan-inoculated	Leaf
8	Day 3	Seosan-inoculated	Root
9	Day 14	Mock	Leaf
10	Day 14	Mock	Root
11	Day 14	Seosan-inoculated	Leaf
12	Day 14	Seosan-inoculated	Root
13	Day 28	Mock	Leaf
14	Day 28	Mock	Root
15	Day 28	Seosan-inoculated	Leaf
16	Day 28	Seosan-inoculated	Root
17	Day 35	Mock	Leaf
18	Day 35	Mock	Root
19	Day 35	Seosan-inoculated	Leaf
20	Day 35	Seosan-inoculated	Root

**Table 4:** List of primer sequences used for qRT-PCR of auxin-related genes.

*Arabidopsis* Homolog (Accession Number)	Gene Name	BRAD ID	cDNA Size (bp)	Primer Forward (F) and Reverse(R)	Product Size (bp)
At2g23170	*BrGH3.1*	Bra039186	1791	AAGGACTTGAAAGCTCTCAG	156
				CTTAAATGTTTTCCGGTCGG	
At1g72540	*BrDCK1*	Bra008038	1344	ATCCAGACAAACCTCTGTTC	177
				AATCCTCCTTCACCGAGATA	
At5g13360	*BrGH3.2*	*Bra008833*	1758	AGTGAGACAGCCTCTAGAAT	177
				TCGTTGTATAAGCCCACAAA	
At4g34760	*BrSAUR1*	Bra011560	324	TTCCACTCGATGTACCAAAG	197
				TGAGGTTAGGGTCTGAAAGA	
At1g77690	*BrLAX1*	*Bra008325*	1419	CTGTGGAGATAATGCATGCCA	161
				GAGTAGAGAGAGTGCATTGG	
At1g73590	*BrPIN1*	Bra015983	1857	GGAAACAGAAAAGCAGCATT	187
				GAGTAGAGAGAGTGCATTGG	
At1g48630	*BrRACK1*	Bra032261	984	ACATGATCGTCACTTCTTCC	243
				AGAGAACGTCTTTCGTGTGT	
At3g12830	*BrSAUR2*	Bra039345	402	GAATCTTTCCTCCGGTCTTC	187
				TTTGCTCGTAACCATACTCC	
At3g44300	*BrNIT1*	Bra028932	1032	GAATCAGAGGGTCTCATCAC	151
				TCGAAACGAATGTAACTGGT	
At5g22300	*BrNIT2*	Bra020184	1074	GCAAGTATCTTGCTTCTGCT	157
				TGACCATGTGAGTCAAAGAA	
At5g01990	*BrPILS1*	Bra014265	1296	GAAGCAAATCATCCAACCTG	167
				GTGCCAGTAATATACAGGGG	
At1g59500	*BrGH3.3*	Bra041046	1764	TAAGCTTCCTCCAGAACAAC	157
				GAGCAAACCTCTACCAAGAA	
At4g14550	*BrIAA1*	Bra039855	702	CTCTACCAAAGAAGGATCCG	163
				TTCGCTACACTTCTGATGAG	
At4g03400	*BrGH3.4*	Bra034205	1833	ATGTTGAAGAGATGGACGAC	208
				CTATGGCAAGTAAACGGAAC	
At3g23050	*BrIAA2*	Bra033886	729	AGCTGCATTGGTTAAGGTAT	184
				CAGATCCATTAGCTTGCTCT	
At4g27260	*BrGH3.5*	Bra019060	1839	AAGAACGTGATGCCTGTTAT	183
				TGATCTCCGGTCTAGTTCTT	
At4g27450	*BrARG10-like*	Bra026348	753	CTCTGACAACACTTTCTCCA	175
				GGTCAGACCATATTGCTTG	
At3g16500	*BrIAA3*	Bra001634	819	GAGGTCAAGTCAACAAGAGT	171
				CTCTGAAGAGTTGGTCTACG	
At2g04850	*BrARP1*	Bra013191	1212	GAATGTGTTCCAAGGGTTTG	189
				CTGCTGCATCTATCATCAGT	
At2g46690	*BrSAUR3*	Bra039264	360	GCTCTGAAAGGCTTCCATTTAC	222
				AACGTCGCATGGAATTGTGATG	
At5g25890	*BrIAA4*	Bra009867	531	TTCGTCGGATCTAAACAGAC	167
				CCTTGCAGTTCTTTCTAGGT	
At1g04240	*BrIAA5*	Bra032521	543	ATCCCCTCCTAGAAAGACTC	152
				TTTTCCTCAAGTATGGTGCA	
At3g60690	*BrSAUR4*	Bra014476	513	ATACCAATTGGGAAGGAACC	172
				CAAACTCTTCCTCTGCTTCT	
XM_009147610.2	*ACTIN1*		1371	F: CAACCAATCGTCTGTGACAA	105
				R: ATGTCTTGGCCTACCAACAA	
FJ969844.1	*ACTIN2*		491	F: AATGGTACCGGAATGGTCAA	119
				R: TCCTTCTGGTTCATCCCAAC	

## Supplementary Materials

The following are available online at https://www.mdpi.com/1422-0067/21/15/5554/s1, Figure S1. Phylogenetic tree showing association between auxin biosynthesis genes (black) in *Arabidopsis thaliana* and orthologues in *Brassica rapa*. Different colour codes of *B. rapa* genes represent different protein/gene families. BrGH3—violet, BrIAA—light blue, BrSAUR—green, BrPIN—orange, BrARG10like—yellow, BrLAX—pink, BrPILS—dark red, BrGH3—light green, BrNIT- chocolate, BrDCK-Red and BrARP—dark blue, Table S1. Protein homology analysis of 23 genes involved in auxin metabolism, signaling, and transport in *Brassica rapa.*
