# Supplementary material for: Expression and Role of Biosynthetic, Transporter, Receptor, and Responsive Genes for Auxin Signaling during Clubroot Disease Development
Source: Int J Mol Sci. 2020 Aug 3;21(15):5554. doi: 10.3390/ijms21155554 (PMC7432499; doi:10.3390/ijms21155554)
Supplement: Supplementary file 1 [file ijms-21-05554-s001.pdf]

Expression and Role of Biosynthetic, Transport, Receptor and Responsive Genes for Auxin Signaling during Clubroot Disease Development

Arif Hasan Khan Robin [1,2,], Gopal Saha [1,3], Rawnak Laila [1,], Jong-In Park [1,], , Hoy-Teak Kim [1] , and Ill-Sup Nou [1,*]

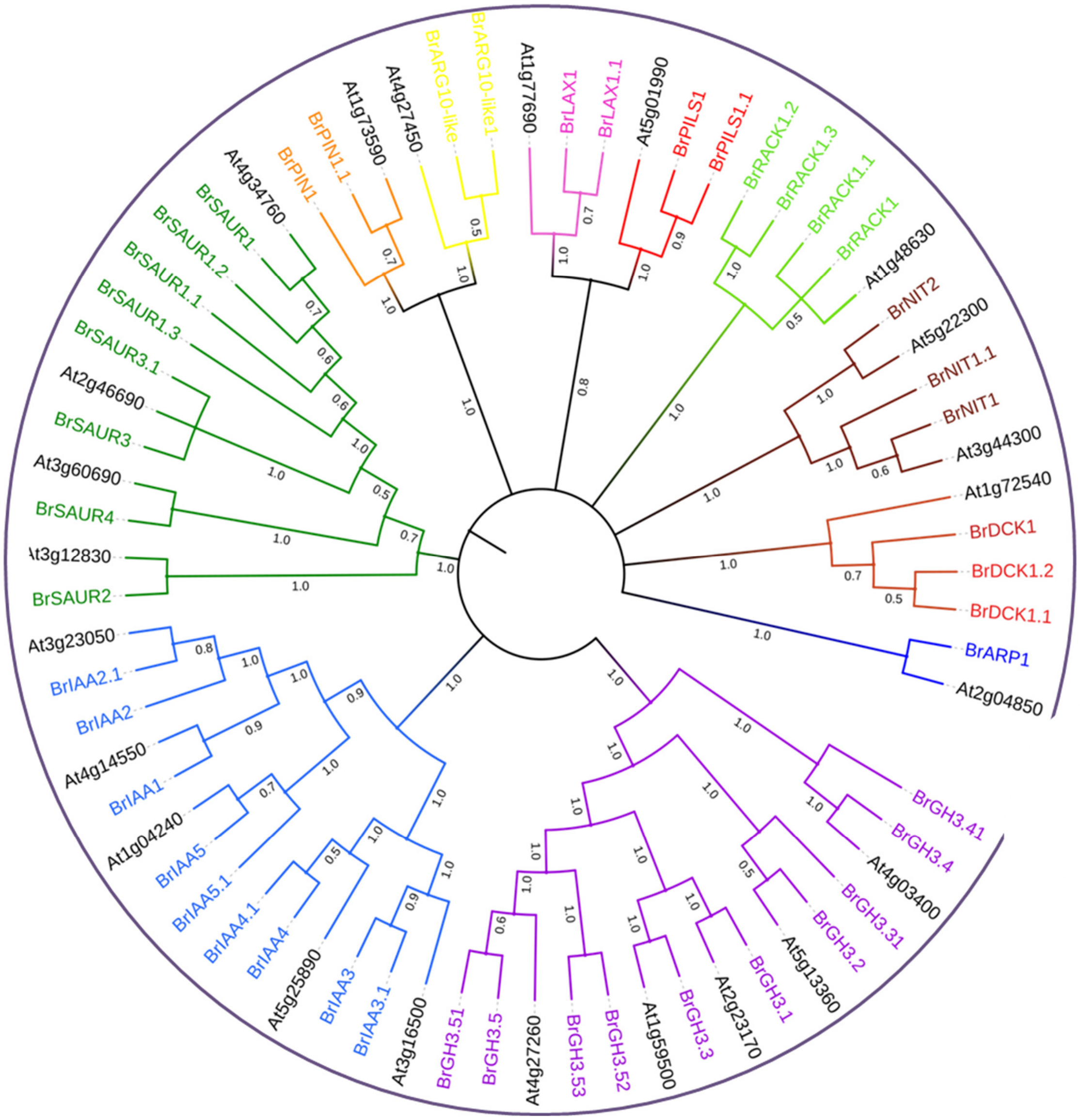

Figure S1. Phylogenetic tree showing association between auxin biosynthesis genes (black) in *Arabidopsis thaliana* and orthologues in *Brassica rapa*. Different colour codes of *B. rapa* genes represent different protein/gene families. BrGH3 – violet, BrIAA – light blue, BrSAUR – green, BrPIN – orange, BrARG10like – yellow, BrLAX – pink, BrPILS – dark red, BrGH3 – light green, BrNIT- chocolate, BrDCK-Red and BrARP – dark blue.

Table S1. Protein homology analysis of 23 genes involved in auxin metabolism, signaling and transport in *Brassica rapa*.

Sl.	Gene Name	Top matched clones	Name of protein	% Identity	E-value	Top homologous species	References
1.	*BrDCK1*	NP_177398	Protein kinase superfamily protein	89.24	0.0	*Arabidopsis thaliana*	Theologis et al., 2000
2.	*BrLAX1*	NP_177892	like AUX1 3	96.82	0.0	*Arabidopsis thaliana*	Theologis et al., 2000
3.	*BrPIN1*	NP_177500	Auxin efflux carrier family protein	90.45	0.0	*Arabidopsis thaliana*	Theologis et al., 2000
4.	*BrRACK1*	NP_175296	Receptor for activated C kinase 1B	92.66	0.0	*Arabidopsis thaliana*	Theologis et al., 2000
5.	*BrGH3.1*	OAP10272	GH3.3	90.94	0.0	*Arabidopsis thaliana*	Zapata et al., 2016
6.	*BrGH3.2*	NP_001190301	Auxin-responsive GH3 family protein	79.29	0.0	*Arabidopsis thaliana*	Institute et al., 2000
7.	*BrGH3.3*	NP_196841	Auxin-responsive GH3 family protein	77.98	0.0	*Arabidopsis thaliana*	Institute et al., 2000
8.	*BrGH3.4*	NP_001319858	Auxin-responsive GH3 family protein	89.36	0.0	*Arabidopsis thaliana*	Mayer et al., 1999
9.	*BrGH3.5*	NP_194456	Auxin-responsive GH3 family protein	96.24	0.0	*Arabidopsis thaliana*	Mayer et al., 1999
10.	*BrIAA1*	NP_193191	indole-3-acetic acid inducible 14	84.21	1e-137	*Arabidopsis thaliana*	Mayer et al., 1999
11.	*BrIAA2*	NP_188945	indole-3-acetic acid 7	88.07	8e-147	*Arabidopsis thaliana*	Salanoubat et al., 2000
12.	*BrIAA3*	NP_188271	phytochrome-associated protein 1	80.00	2e-153	*Arabidopsis thaliana*	Salanoubat et al., 2000
13.	*BrIAA4*	NP_568478	indole-3-acetic acid inducible 28	88.14	5e-96	*Arabidopsis thaliana*	Institute et al., 2000
14.	*BrIAA5*	NP_001322162	AUX/IAA transcriptional regulator family protein	83.07	3e-108	*Arabidopsis thaliana*	Theologis et al., 2000
15.	*BrSAUR1*	NP_195202	SAUR-like auxin-responsive protein family	96.26	6e-72	*Arabidopsis thaliana*	Mayer et al., 1999
16.	*BrSAUR2*	NP_187889	SAUR-like auxin-responsive protein family	87.12	2e-76	*Arabidopsis thaliana*	Salanoubat et al., 2000
17.	*BrSAUR3*	NP_182192	SAUR-like auxin-responsive protein family	86.78	2e-69	*Arabidopsis thaliana*	Lin et al., 1999
18.	*BrSAUR4*	NP_191628	SAUR-like auxin-responsive protein family	85.29	7e-103	*Arabidopsis thaliana*	Salanoubat et al., 2000
19.	*BrNIT1*	NP_190016	nitrilase 2	85.50	0.0	*Arabidopsis thaliana*	Salanoubat et al., 2000
20.	*BrNIT2*	6I00_A	Chain A, Bifunctional nitrilase/nitrile hydratase NIT4	88.95	0.0	*Arabidopsis thaliana*	Mulelu et al., 2019
21.	*BrPILS1*	NP_195819	Auxin efflux carrier family protein	89.17	0.0	*Arabidopsis thaliana*	Institute et al., 2000
22.	*BrARG10-like*	NP_567775	aluminum induced protein with YGL and LRDR motifs	95.60	2e-178	*Arabidopsis thaliana*	Mayer et al., 1999
23.	*BrARP1*	NP_565316	Auxin-responsive family protein	92.54	0.0	*Arabidopsis thaliana*	Lin et al., 1999

References

Institute, K.D.R.; Harbor, T.C.S. Washington University Sequencing Consortium and European Union Arabidopsis Genome Sequencing Consortium, Sequence and analysis of chromosome 5 of the *plant Arabidopsis thaliana*. *Nature* **2000**, 408(6814), p.823.

Lin, X.; Kaul, S.; Rounsley, S.; Shea, T.P.; Benito, M.I.; Town, C.D.; Fujii, C.Y.; Mason, T.; Bowman, C.L.; Barnstead, M.; Feldblyum, T.V. Sequence and analysis of chromosome 2 of the plant *Arabidopsis thaliana*. *Nature* **1999**, 402(6763), pp.761-768.

Mayer, K.; Schüller, C.; Wambutt, R.; Murphy, G.; Volckaert, G.; Pohl, T.; Düsterhöft, A.; Stiekema, W.; Entian, K.D.; Terryn, N.; Harris, B.; Sequence and analysis of chromosome 4 of the plant *Arabidopsis thaliana*. *Nature* **1999**, 402(6763), pp.769-777.

Mulelu, A.E.; Kirykowicz, A.M.; Woodward, J.D., Cryo-EM and directed evolution reveal how *Arabidopsis* nitrilase specificity is influenced by its quaternary structure. *Comm. Biol*. **2019,** 2(1), pp.1-11.

Salanoubat, M.; Lemcke, K.; Rieger, M.; Ansorge, W., European Union chromosome 3 *Arabidopsis* sequencing Consortium; Institute for genomic research; Kazusa DNA research Institute. *Nature* **2000**. 408, 820-822.

Zapata, L.; Ding, J.; Willing, E.M.; Hartwig, B.; Bezdan, D.; Jiao, W.B.; Patel, V.; James, G.V.; Koornneef, M.; Ossowski, S; Schneeberger, K. Chromosome-level assembly of *Arabidopsis thaliana* L reveals the extent of translocation and inversion polymorphisms. **2016**, *Proceed. Nat. Aca. Sci.* **113**(28), pp.E4052-E4060.
